# Greater Social Interest Between Autistic and Non-autistic Conversation Partners Following Autism Acceptance Training for Non-autistic People

**DOI:** 10.3389/fpsyg.2021.739147

**Published:** 2021-09-22

**Authors:** Desiree R. Jones, Kerrianne E. Morrison, Kilee M. DeBrabander, Robert A. Ackerman, Amy E. Pinkham, Noah J. Sasson

**Affiliations:** Department of Psychology, School of Behavioral and Brain Sciences, The University of Texas at Dallas, Richardson, TX, United States

**Keywords:** first impressions, inclusion, stigma, intervention, double empathy problem

## Abstract

Bi-directional differences in social communication and behavior can contribute to poor interactions between autistic and non-autistic (NA) people, which in turn may reduce social opportunities for autistic adults and contribute to poor outcomes. Historically, interventions to improve social interaction in autism have focused on altering the behaviors of autistic people and have ignored the role of NA people. Recent efforts to improve autism understanding among NA adults via training have resulted in more favorable views toward autistic people, yet it remains unknown whether these benefits extend to real-world interactions between autistic and NA people. The current study explores whether a brief autism acceptance training (AAT) program can improve social interactions between autistic and NA adults. Thirty-nine NA males were randomly assigned to complete AAT or a no-training control condition, then participated in a 5-min unstructured conversation with an unfamiliar autistic male (*n* = 39). Following the conversation, participants rated their perceptions of interaction quality, first impressions of their partner, and their interest in future interactions with their partner. In dyads where the NA individual completed AAT, both the autistic and NA person endorsed greater future interest in hanging out with their partner relative to dyads in which the NA adult did not complete AAT. However, other social interaction outcomes, including ratings of interaction quality and first impressions of autistic partners, largely did not differ between training and no-training conditions, and assessments of the interaction were largely unrelated for autistic and NA partners within dyads. Results also indicated that NA participants, but not autistic participants, demonstrated substantial correspondence between evaluations of their partner and the interaction, suggesting that autistic adults may place less weight on trait judgments when assessing the quality of an interaction. These findings suggest that the brief AAT for NA adults used in this study may increase mutual social interest in real-world interactions between NA and autistic adults, but more systematic changes are likely needed to bridge divides between these individuals. Future work with larger, more diverse samples is recommended to further explore whether interventions targeting NA adults are beneficial for improving autistic experiences within NA social environments.

## Introduction

Difficulties with social interactions are common for autistic adults. They report few close friendships (Howlin et al., 2000; Orsmond et al., 2004) and are more likely to experience social exclusion and low quality of life compared to adults with cognitive or other developmental disabilities (Orsmond et al., 2013; DaWalt et al., 2019). These outcomes are even found for autistic adults without intellectual disability (Farley et al., 2009; Howlin and Moss, 2012; Lord et al., 2020), and appear largely independent of a person's autistic traits (Magiati et al., 2014). In fact, autistic people commonly experience similar, or even worsening, social disability as adults despite a measured reduction in autistic traits from childhood to adulthood (Howlin et al., 2013). Although previous work has primarily attributed interpersonal difficulties in autism to intrinsic deficits in social cognition and behavior (Baron-Cohen et al., 1985; Oberman et al., 2005), more recent empirical advances (Sasson et al., 2017; Morrison et al., 2019a; Crompton et al., 2020b), and informed expertise from autistic people (Yergeau, 2013; Milton and Sims, 2016; Gillespie-Lynch et al., 2017; Kapp, 2019; Raymaker et al., 2020), have increasingly highlighted the role of bi-directional factors, including inhospitable social environments, and the behaviors of non-autistic (NA) people, that also contribute to poor social experiences for autistic people (Milton et al., 2013; Sasson et al., 2017; Morrison et al., 2019a; Crompton et al., 2020b).

This reframing of interpersonal difficulties in autism from individual to relational is exemplified by The Double Empathy Problem (DEP; Milton, 2012). The DEP eschews traditional deficit-model explanations for the social difficulties autistic people often experience in favor of a transactional explanation, driven by a mutual breakdown of communication between people with different modes of social communication and understanding. In contrast with the decades of research documenting autistic difficulties inferring the mental states, emotions, and intentions of NA individuals (Baron-Cohen et al., 1985; Schultz, 2005; Morrison et al., 2019b), a growing empirical literature grounded in the DEP framework has found that NA adults make similar social cognitive errors when trying to understand their autistic peers (Edey et al., 2016; Sheppard et al., 2016). These misperceptions can lead NA adults to view autistic people unfavorably (Alkhaldi et al., 2019), and may contribute to social exclusion and poor mental health among autistic adults (Mitchell et al., 2021).

A bi-directional difference in communication styles for autistic and NA adults is further supported by differences in interaction outcomes for mixed vs. matched neurotype interactions. Within dyadic interactions consisting of either two autistic adults, two NA adults, or an autistic adult paired with a NA adult, both autistic and NA individuals showed a greater interest in future interactions with individuals who shared their neurotype (Morrison et al., 2020). Qualitative reports from autistic adults suggest that this preference may relate to an increased understanding and acceptance of autistic communication styles in interactions between autistic individuals (Crompton et al., 2020a). Indeed, a study of information transfer between autistic and NA adults (Crompton et al., 2020b) found that chains of alternating autistic and NA adults experienced greater communication difficulty than chains consisting entirely of autistic or NA individuals, which did not differ from each other. Collectively, these findings highlight the ways in which a mismatch between autistic and NA communication styles can impact autistic-NA interactions, and suggest that the “fault” of interaction difficulties between autistic and NA partners does not lie with either person alone, but in the intersection between the two.

Traditional deficit-model frameworks of autistic interaction difficulties have almost exclusively centered treatment on the autistic person via social skills and social cognitive training, with the implicit assumption that teaching more normative modes of social understanding and behavior will translate into improved social outcomes. These interventions have generally failed to produce lasting benefits for autistic adults (Bottema-Beutel et al., 2018), and may unintentionally encourage the masking of autistic ways of being (Pearson and Rose, 2021), increase internalized stigma (Botha and Frost, 2020), and contribute to depression (Cage et al., 2018), anxiety (Hull et al., 2021), and even suicidality (Cassidy et al., 2020) in adulthood. Furthermore, because many autistic individuals consider autism to be central to their identity (Botha et al., 2020; Crompton et al., 2020a), interventions designed to alter their core characteristics have been criticized as unnecessary or even abusive (Milton, 2014; Kirkham, 2017; McGill and Robinson, 2020).

Therefore, given that deficit-model treatments for social disability among autistic adults are minimally effective at improving life outcomes, and may in some cases harm mental well-being, alternative approaches for improving interpersonal difficulties between autistic and NA adults are beginning to be considered and tested (Jones et al., 2021). One potential avenue capitalizes on recent findings suggesting that improving autism knowledge and acceptance among NA individuals shows promise for reducing biases toward autistic children and adults (Gillespie-Lynch et al., 2015; Dickter et al., 2020a) and increasing inclusive attitudes (Jones et al., 2021). However, the benefits of autism training may not extend to all forms of bias (Dickter et al., 2020a; Bast et al., 2021; Jones et al., 2021), and it is unknown whether previously reported benefits translate beyond experimental settings to real-world interactions between autistic and NA individuals.

In our previous study (Jones et al., 2021), NA participants viewed a brief autism acceptance training (AAT) video, then rated their first impressions of videos of autistic adults, answered questionnaires assessing their autism stigma, perceptions of autistic abilities, and autism knowledge, and completed an implicit association test (IAT) to measure their implicit biases about autism. We found that compared to adults who completed a general mental-health focused training, as well as those in a no-training condition, participants who completed AAT had more positive perceptions of autistic abilities, greater interest in interacting with autistic individuals, and less autism stigma. However, implicit biases did not differ significantly across training conditions, suggesting that the training may have a limited impact on more subtle or covert forms of bias. The current study seeks to expand our previous work to a real-world setting to evaluate whether a brief AAT module for NA adults can lead to more positive interactions between autistic and NA adults. Non-autistic adults were assigned to either an AAT condition or a no-training control condition, with participants in the AAT condition initially viewing a 25-min video featuring factual information about autism and firsthand accounts from autistic adults (Jones et al., 2021). Non-autistic participants across both conditions were then paired with an unfamiliar autistic adult and completed a 5-min unstructured dyadic interaction, with participants blinded to their partner's diagnosis. Following the interaction, participants responded to questionnaires assessing their impressions of both the interaction and their interaction partner. Based on our previous findings (Jones et al., 2021), we predicted that NA participants in the AAT condition would rate their autistic partners more favorably and would have greater interest in interacting with them compared to NA participants in the control condition. At the level of the interaction, we predicted that NA participants in the AAT condition would feel closer to their autistic partners and would rate the interaction as higher quality compared to NA participants in the control condition. Because the training was designed to target the NA person, hypotheses centered on their responses to autistic partners, but the employed dyadic analyses also examined whether autistic adults evaluated NA adults who completed the training more favorably than those who did not. If supported, these hypotheses would provide evidence for the use of this training as a brief, accessible tool to improve interactions between autistic and NA adults.

## Methods

### Participants

Autistic and NA participants (*N* = 80) were young adult males recruited from The University of Texas at Dallas, the local community, and word of mouth. Participants were approximately matched on race, age, and scheduling availability to form dyads, with each dyad consisting of one autistic adult and one NA adult. Inclusion was restricted to males to limit the influence of gender on interaction dynamics. All autistic participants were administered the Autism Diagnostic Observation Schedule, Second Edition (ADOS-2; Lord et al., 1989), and those not meeting the cutoff for autism spectrum disorder were excluded from participation, as were those with an approximated IQ score below 80 as estimated by the reading subscale of the Wide Range Achievement Test, Third Edition (WRAT-3; Wilkinson, 1993), a brief assessment that correlates highly with full-scale IQ scores (Powell et al., 2002). Exclusion criteria for NA participants consisted of a self-reported diagnosis of autism or a developmental disability, and/or an estimated IQ under 80 based on the WRAT-3. A total of 40 autistic males and 40 NA males ages 18-27 (*M* = 20.46; SD = 1.75) participated in the study. However, one autistic participant failed to meet inclusion criteria and their dyad was therefore excluded from analysis, resulting in a final total of 78 participants across 39 dyads.

Participant demographics are reported in Table 1. Overall, autistic and NA participants did not differ significantly on age [*t*_(76)_ = −0.19, *p* = 0.849], race [$\chi_{\left(2\right)}^{2}$ = 2.67, *p* = 0.434], ethnicity [$\chi_{\left(2\right)}^{2}$ = 1.84, *p* = 0.310], or WRAT-3 IQ [*t*_(76)_ = −1.72, *p* = 0.090]. However, within dyads, WRAT-3 IQ scores were significantly lower in autistic individuals than their NA partners [*F*_(1, 37)_ = 4.43, *p* = 0.042] and were therefore covaried in analyses.

**Table 1:** Participant demographics by training condition and diagnosis.

	**Autism acceptance training (** ***N*** **= 19 dyads)**		**Control (** ***N*** **= 20 dyads)**	
	**Autistic (*N* = 19)**	**NA (*N* = 19)**	**Autistic (*N* = 20)**	**NA (*N* = 20)**
**Race**				
White	84%	90%	95%	89%
Asian	5%	10%	5%	11%
Bi/Multiracial	11%	0%	0%	0%
**Ethnicity**				
Hispanic/Latino	5%	5%	10%	30%
Age [M (SD)]	20.26 (2.08)	20.26 (1.49)	20.55 (1.47)	20.70 (2.03)
WRAT-3 IQ	109.89 (10.62)	113.47 (7.29)	108.80 (12.33)	112.75 (8.15)

*WRAT-3, Wide Range Achievement Test 3*.

### Procedure

Dyads were assigned to either the AAT condition or a control condition. As part of informed consent procedures, all participants were told that they would be participating in a study about social interactions, and that they would be interacting with a stranger who, “may or may not be autistic.” Autistic participants were not discouraged from disclosing their diagnosis, but only two participants in the study chose to do so (one in each condition). Before beginning the dyadic portion of the study, NA participants in the AAT condition watched a narrated 25-min AAT module (Jones et al., 2021). This training features firsthand accounts from autistic adults, as well as information on autistic strengths, neurodiversity, sensory sensitivities, and ways to promote inclusion and acceptance of autism among college students. In a previous study of NA adults (Jones et al., 2021), the use of this training was associated with more inclusive attitudes toward autistic adults and fewer misconceptions about autism, when compared to a more general mental-health focused training and a no-training control condition. Autistic participants, as well as NA participants in the control condition, did not receive any training. All other study procedures were consistent across participants.

Participants were seated across from one another to complete a 5-min, unstructured conversation previously used with autistic (Morrison et al., 2020), NA (Berry and Hansen, 1996), and mixed dyads (Usher et al., 2018; Morrison et al., 2020). Participants were instructed to speak freely for the full 5 min with the goal of getting to know one another, and conversations were videotaped. To avoid the potential effect of demand characteristics, the participants were not given information about their partner's diagnostic status. Following the interaction, each participant completed computerized questionnaires in a counterbalanced order that assessed their perceptions of the interaction quality, their partner, and their feelings of closeness, followed by a brief demographics questionnaire. Participants were then administered the WRAT-3 reading subtest (Wilkinson, 1993). Participants were compensated for their time with either $50 or course credit. All study procedures were approved by the university's Institutional Review Board.

### Measures

#### The Social Interaction Evaluation Measure

The Social Interaction Evaluation Measure (Berry and Hansen, 1996) is an 11-item Likert-type scale used to evaluate interaction quality (Berry and Hansen, 1996). Participants rated items reflecting their perceptions of both the interaction (e.g., “how much did you enjoy the interaction,” “to what extent was the interaction intimate”) and the partner's role in the interaction (e.g., “how much did your partner disclose in the interaction,” “how much did your partner influence the conversation”) on a scale of 1–8, with higher scores indicating more positive evaluations. Scores on each item were averaged to create a composite score representing interaction quality. This measure has demonstrated validity for observer ratings of interaction quality (Berry and Hansen, 1996) and has been used successfully when assessing interactions in autism (Morrison et al., 2020). Within the present sample, this measure demonstrated acceptable internal consistency (autistic group α = 0.72; NA α = 0.73).

#### The Subjective Closeness Index and The Subjective Closeness Index

The Subjective Closeness Index (Berscheid et al., 1989) and the Inclusion of the Other in the Self (Aron et al., 1992) assess a participant's feelings of “closeness” to their partner. For the Subjective Closeness Index (Berscheid et al., 1989), participants rated their perceived closeness with their partner on two Likert-type items. Possible total scores range from 2 to 14, with higher scores indicating greater perceived closeness. For the Inclusion of the Self in the Other (Aron et al., 1992), participants were presented with pairs of increasingly overlapping circles and asked to choose the pair best representing how close they felt with their partner. Scores range from 1 to 7, with 1 indicating no overlap with the other individual and 7 indicating high overlap. Based on previous analyses (Aron et al., 1997; Morrison et al., 2020), a composite score was created by averaging the raw scores of these two scales, resulting in an overall metric of closeness. Previous research has shown strong psychometric properties for this combined scale (Aron et al., 1997; Morrison et al., 2020). In our sample, this measure demonstrated good internal consistency (AUT α = 0.81, NA α = 0.85).

#### The International Personality Item Pool-Interpersonal Circumplex

The International Personality Item Pool-Interpersonal Circumplex (IPIP-IPC; Markey and Markey, 2009) is a 32-item questionnaire used to evaluate a participant's assessment of their partner's warmth and dominance, two factors that predict quantity, and quality of social interaction (Wiggins, 1982; McCrae and Costa, 1989; Horowitz et al., 2006). Participants rated their partner on a five-point Likert-type scale for items assessing interpersonal warmth (e.g., “My partner reassures others”) and dominance (e.g., “My partner speaks loudly”), with higher scores indicating greater agreement with each item. Items were divided into octants, each containing four items, with octant scores based on the average score of these four items. Octant scores were then used to create indices of interpersonal dominance and warmth ratings attributed to the conversation partner. This measure correlates highly with behavioral indices of warmth and dominance and shows strong psychometric properties in both the general population and autistic adults (Markey and Markey, 2009; Morrison et al., 2020).

#### The First Impressions Scale

The First Impressions Scale (Sasson et al., 2017) is a 10-item scale designed to assess a rater's initial impressions of a target individual. Six items reflect perceptions of personal traits (awkwardness, attractiveness, dominance, trustworthiness, likeability, and intelligence), while the remaining four items reflect “behavioral intent,” or the rater's interest in future interactions with the target individual across different contexts. For each item, participants rated their interaction partner on a four-point Likert-type scale. This scale has previously been used to evaluate perceptions of autistic adults by both autistic and NA raters (Sasson et al., 2017; DeBrabander et al., 2019) and has recently been used for evaluations of in-person interactions between autistic and NA adults (Morrison et al., 2020).

### Analysis Plan

Zero-order correlations between participants' interaction ratings were evaluated to assess the relationship between these indicators, as well as the consistency of ratings between partners. To account for unequal variances between autistic and NA participants, two factor mixed-model ANOVAs were run using a Greenhouse-Geisser correction, assessing the effects of diagnosis (autistic vs. NA) and training condition (AAT vs. control) on how participants evaluated their conversation partner and the overall interaction. Specifically, training condition was treated as a between-subjects variable and autistic and NA interaction ratings (interaction quality, first impressions, closeness, warmth and dominance) were treated as a within-subjects factor, with separate analyses run for each outcome measure. As IQ differed significantly between autistic and NA individuals within dyads, WRAT-3 scores were included as a covariate in each ANOVA. All analyses were completed using SPSS 27 (IBM SPSS Inc., 2015).

## Results

### Correlations Between Ratings

Zero-order correlations to assess the relationships between interaction ratings in autistic and NA participants are reported in Table 2. While interaction quality reported by autistic adults only correlated significantly with their ratings of closeness, higher ratings of interaction quality reported by NA participants correlated with greater closeness and many other factors as well, including higher ratings of their autistic partner being likable, intelligent, and warm, lower ratings of them being awkward, and increased interest in hanging out with and starting conversations with them. Non-autistic participants who rated their autistic partners as more intelligent also endorsed a stronger desire to hang out with, sit near, and have a conversation with them, while autistic participants' ratings of their partner's intelligence were not significantly correlated with any of their other ratings. Perceptions of the partner's intelligence correlated significantly with the partner's measured intelligence for NA participants rating autistic partners (*r* = 0.378, *p* = 0.018), but did not reach significance for autistic participants rating NA partners (*r* = 0.244, *p* = 0.135). In both groups, the desire to sit near, hang out with, and have a conversation with the partner were all moderately correlated with one another. In the NA but not autistic group, the desire to hang out with and have a conversation with the autistic partner were also associated with greater feelings of closeness.

**Table 2:** Zero-order correlations between social interaction ratings, with correlations for ratings made by autistic participants above the diagonal and those made by non-autistic participants below it.

	**Awkward**	**Attractive**	**Trustworthy**	**Dominant**	**Likable**	**Intelligent**	**Live near**	**Hang out**	**Sit near**	**Conversation**	**Closeness**	**IPIP-IPC warmth**	**IPIP-IPC dominance**	**Interaction quality**
Awkward	1	−0.278	−0.148	0.085	−0.083	−0.148	0.277	−0.140	0.149	0.103	0.179	−0.193	−0.251	−0.017
Attractive	–**0.394**	1	**0.319**	0.101	0.174	0.152	–**0.445**	−0.081	−0.046	0.057	−0.104	−0.015	**0.348**	−0.201
Trustworthy	0.037	0.109	1	−0.124	0.128	0.297	−0.090	−0.007	0.007	−0.034	0.073	0.085	0.195	−0.154
Dominant	−0.201	0.002	0.106	1	−0.194	−0.267	0.026	−0.127	−0.158	−0.032	−0.118	–**0.327**	0.154	0.093
Likable	0.077	0.238	0.049	−0.247	1	0.097	0.044	0.164	0.000	−0.066	−0.087	−0.044	0.023	0.125
Intelligent	–**0.409**	0.276	0.275	−0.123	0.134	1	−0.114	0.068	0.038	0.118	0.241	−0.017	0.006	0.046
Live near	0.162	−0.096	−0.055	−0.116	0.184	−0.164	1	0.105	0.239	0.127	0.107	−0.015	−0.216	0.250
Hangout	–**0.494**	0.088	0.149	0.097	0.030	**0.404**	0.043	1	**0.431**	**0.653**	0.264	0.099	−0.070	0.219
Sit near	−0.167	0.123	0.010	0.064	**0.419**	**0.381**	0.245	**0.417**	1	**0.649**	0.169	−0.021	−0.031	0.126
Conversation	–**0.365**	0.015	0.102	0.118	0.254	**0.453**	0.101	**0.535**	**0.458**	1	0.282	−0.099	−0.118	−0.042
Closeness	−0.204	0.000	0.198	0.148	0.172	0.311	−0.084	**0.352**	0.285	**0.435**	1	−0.168	0.083	**0.343**
IPIP-IPC warmth	–**0.500**	**0.432**	0.174	−0.095	0.216	0.100	0.000	0.227	0.082	0.193	0.068	1	0.307	0.072
IPIP-IPC Dominance	−0.206	0.042	−0.293	0.283	0.100	0.046	0.185	0.279	**0.343**	0.315	0.220	0.262	1	0.167
Interaction Quality	–**0.490**	0.120	0.094	−0.068	**0.327**	**0.367**	0.054	**0.403**	0.204	**0.612**	**0.550**	**0.351**	0.263	1

*Bold values signify significance at the level of p < 0.05. Values reflect an individual's evaluations of the interaction and their conversation partner. IPIP-IPC, International Personality Item Pool-Interpersonal Circumplex*.

Correlations and covariances between ratings given by autistic participants and their NA partners within dyads are reported in Table 3. Overall, outcome ratings were generally unrelated between partners. However, there was a negative correlation between ratings of warmth (*r* = −0.358, *p* = 0.025), with participants whose partners rated them as higher in warmth in turn rating their partners as less warm.

**Table 3:** Zero-order correlations and covariances between social interaction ratings for autistic and non-autistic participants within dyads.

	**Awkward**	**Attractive**	**Trustworthy**	**Dominant**	**Likable**	**Intelligent**	**Live Near**	**Hang Out**	**Sit near**	**Conversation**	**Closeness**	**IPIP-IPC warmth**	**IPIP-IPC dominance**	**Interaction quality**
Correlation	−0.113	0.005	−0.138	−0.152	0.277	0.087	−0.065	0.039	0.207	−0.056	−0.044	–**0.358**	0.142	0.087
Covariance	−0.049	0.002	−0.020	−0.040	0.070	0.049	−0.039	0.014	0.103	−0.018	−0.050	−0.190	0.134	0.042

*Bold values signify significance at the level of p < 0.05. Values reflect an individual's evaluations of the interaction and their conversation partner. IPIP-IPC, International Personality Item Pool-Interpersonal Circumplex*.

### Social Interaction Measures

Means and standard deviations for all social interaction measures are reported in Table 4 and fixed effects for the impacts of training condition, participant diagnosis, and their interaction on these ratings are reported in Table 5. For indicators of interaction quality, there was a significant main effect of training condition on intention to hang out with the partner, with both autistic and NA participants in the AAT condition reporting a stronger intention to hang out with their partner in their free time [*F*_(1, 35)_ = 6.60, *p* = 0.015, partial η^2^ = 0.159]. Participants in the training condition rated their partners as less trustworthy compared to those in the control condition [*F*_(1, 35)_ = 4.99, *p* = 0.032, partial η^2^ = 0.125]. Ratings on these items did not differ significantly as a function of actor diagnosis or the interaction between training condition and diagnosis. No significant effects for diagnosis, training condition, or their interaction were found for the IPIP-IPC, closeness, interaction quality, or the remaining first impressions items.

**Table 4:** Means and standard deviations of partner ratings.

	**Autism acceptance training** **(** ***N*** **= 39)**		**Control** **(** ***N*** **= 40)**	
	**Autistic ratings of NA partners**	**NA ratings of autistic partners**	**Autistic ratings of NA partners**	**NA ratings of autistic partners**
	**[M (SD)]**	**[M (SD)]**	**[M (SD)]**	**[M (SD)]**
IPIP-IPC warmth	0.06 (0.78)	−0.15 (0.78)	0.20 (0.82)	−0.21 (0.57)
IPIP-IPC dominance	−0.11 (0.96)	0.30 (1.15)	0.02 (0.81)	−0.23 (0.97)
Closeness	2.76 (1.09)	2.74 (1.12)	2.60 (1.04)	2.68 (1.09)
Interaction quality	5.53 (0.73)	5.42 (0.69)	5.42 (0.59)	5.14 (0.78)
First impressions				
Awkward	3.11 (0.66)	2.63 (0.68)	3.35 (0.67)	2.45 (0.61)
Attractive	2.58 (0.69)	2.26 (0.73)	2.65 (0.59)	2.15 (0.67)
Dominant	1.74 (0.56)	2.00 (0.47)	1.65 (0.59)	1.75 (0.44)
Likable	3.42 (0.51)	3.26 (0.45)	3.25 (0.44)	3.40 (0.59)
Intelligent	3.21 (0.79)	3.05 (0.71)	2.90 (0.72)	3.10 (0.79)
Trustworthy	3.05 (0.41)	3.05 (0.23)	3.25 (0.44)	3.20 (0.41)
Live near	3.00 (0.88)	3.05 (0.85)	2.75 (0.79)	3.15 (0.59)
Hang out	3.05 (0.62)	2.74 (0.45)	2.65 (0.67)	2.45 (0.61)
Sit near	3.26 (0.65)	3.26 (0.87)	3.05 (0.61)	3.10 (0.72)
Conversation	3.16 (0.60)	2.95 (0.62)	2.95 (0.51)	2.75 (0.55)

*Values reflect an individual's evaluations of the interaction and their conversation partner. Higher ratings indicate more positive evaluations. NA, non-autistic; IPIP-IPC, International Personality Item Pool-Interpersonal Circumplex*.

**Table 5:** Fixed effects of training condition, actor diagnosis, and interaction on social interaction measures.

	**Main effect of training condition**		**Main effect of actor diagnosis**		**Interaction**	
	**F**	***p***	**F**	***p***	**F**	***p***
Awkward	0.11	0.74	1.44	0.24	1.71	0.20
Attractive	0.07	0.79	0.81	0.37	0.31	0.58
Dominant	2.41	0.13	0.67	0.42	0.45	0.51
Likable	0.02	0.90	0.47	0.50	2.66	0.11
Intelligent	0.35	0.56	0.03	0.86	1.45	0.24
Trustworthy	**4.99**	**0.03**	1.56	0.22	0.03	0.86
Live near	0.12	0.73	0.85	0.36	0.80	0.38
Hang out	**6.60**	**0.02**	0.51	0.48	0.24	0.62
Sit near	1.02	0.32	2.36	0.13	0.07	0.79
Conversation	1.34	0.23	1.90	0.18	0.01	0.91
IPIP-Warmth	0.18	0.68	0.02	0.88	0.26	0.62
IPIP-Dominance	0.71	0.41	0.28	0.60	2.39	0.13
Closeness	0.18	0.68	2.32	0.14	0.11	0.75
Interaction Quality	1.13	0.28	1.35	0.25	0.23	0.64

*Items reflect participants' ratings of their conversation partner within autistic-non-autistic dyads. Bold values represent significance at p < 0.05*.

To control for the impact of participant IQ on interaction ratings, WRAT-IQ scores were used as a covariate. Within dyads, the WRAT-3 IQ score of the autistic participant, but not the NA participant, was a significant covariate for perceived awkwardness. When comparing across training conditions, autistic WRAT-3 IQ contributed significantly to both partners' ratings for awkwardness, trust, intelligence, and warmth, as well as the intention to hang out with the partner. All other ratings were not significantly predicted by WRAT-3 IQs of autistic or NA participants. Statistical significance did not change for any reported results when IQ was removed as a covariate.

## Discussion

Although previous research has demonstrated that training programs designed to increase autism acceptance and knowledge among NA people can reduce biases and improve inclusive attitudes toward autistic people (Gillespie-Lynch et al., 2015; Dickter et al., 2020a; Jones et al., 2021), no study to date has investigated whether training benefits extend to real-world interactions between autistic and NA people. The current study examines whether an AAT module previously shown to reduce autism stigma among NA adults and increase their interest in interacting with autistic adults presented in videos (Jones et al., 2021) produces improvements to interaction quality and partner evaluation during actual conversations between unfamiliar autistic and NA adults.

Compared to a no-training control condition, both autistic and NA adults reported greater social interest in one another following a “get to know you” conversation when the NA adult had completed AAT. Specifically, both autistic and NA participants in the AAT conditions expressed an increased desire to hang out with their partner in the future, suggesting that AAT not only improved NA adults' social interest in their autistic partners, but also increased their perceived social desirability among autistic participants. Thus, an acceptance training focused solely on NA participants produced a relational effect, leading to social improvements for both partners. Importantly, this improvement occurred despite participants' unawareness of their partner's diagnosis, suggesting that it was not influenced by demand characteristics. This result replicates a previously observed effect of AAT, in which the training increased NA adults' interest in hanging out with autistic people viewed in video clips (Jones et al., 2021), but extends it to real-world interactions with autistic people and, importantly, suggests it may also transfer to increased social interest among autistic adults in their NA partners. Although this result indicates that a brief and relatively easy-to-administer training for NA adults may increase mutual social interest among unfamiliar NA and autistic adults, it remains unclear whether the effect would produce sustained contact and relationship development beyond the experimental session. It is also unknown what, if any, aspects of NA behavior and communication differed following AAT and contributed to increases in social interest. Future work is encouraged to attempt to both replicate this effect and measure whether and how training alters NA behavior within interactions with autistic people. Research examining the impact of AAT on social interactions is limited, but additional training with a greater focus on autistic communication and expressivity may improve NA understanding of neurodivergent interaction styles. Because NA adults have been found to misinterpret autistic communication styles (Brewer et al., 2016; Edey et al., 2016), resulting in a breakdown in communication (Crompton et al., 2020b), how a double empathy focused training may affect perceptions of interaction quality for autistic and NA adults is worthy of further examination.

In contrast to this finding, no effects of training were found on the other three behavioral intention items. However, these items—living near, sitting near, or having a conversation with the person in the future—represent relatively superficial forms of social interaction that can occur with acquaintances or even strangers (Morgan, 2009), whereas the intention to “hang out with” and spend one's free time with another person reflects a closer level of contact associated with the development of friendships (Hays, 1989; Sias and Cahill, 1998), and may be a strong indicator of intimacy, particularly among males (Wood and Inman, 1993; Floyd, 1995; Floyd and Parks, 1995). Autistic individuals often have limited social opportunities (Lord et al., 2020), and can experience difficulties forming friendships (Mazurek, 2014), in part due to how they are perceived by others (Sasson et al., 2017). By increasing interest in future close interactions between autistic and NA adults, AAT may offer potential for improving social opportunities for autistic adults within NA environments.

Independent of training effects, several differences emerged between NA and autistic participants in their evaluations of each other and the interaction. For NA participants, positive ratings of their partner on many first impression and interaction items were associated with higher ratings of interaction quality. In contrast, interaction quality was largely unrelated to how autistic participants evaluated their NA partner. Similarly, NA participants but not autistic participants who perceived their partner to have greater intelligence in turn showed greater social interest in them. This may suggest a greater connection between person and interaction evaluation for NA compared to autistic adults. Such an interpretation is consistent with prior research showing stronger associations between trait evaluation and social interest among NA than autistic people (DeBrabander et al., 2019), and may indicate that trait judgments like awkwardness, likeability, and attractiveness are less relevant to autistic adults than NA adults when judging interaction quality. This interpretation—that autistic individuals place less weight on surface-level traits of their partner when evaluating interactions—is also supported by previous literature suggesting that shared interests rather than individual traits are more of a primary driver of successful friendships for autistic adults (Sosnowy et al., 2019). Future studies investigating other differences in interaction and friendship preferences between NA and autistic adults may highlight additional sources of relational disconnect that, through awareness and understanding, may offer avenues for improving interactions, inclusion, and social outcomes for autistic people.

This interpretation is further supported by the dissociation in ratings made about one another by autistic and NA partners within dyads. Only ratings of warmth significantly correlated between partners, and this correlation was negative, suggesting a disconnect between NA and autistic people in a fundamental aspect of interpersonal assessment. These results are notably different from those found in a previous study of real-world interaction among and between autistic and NA adults using the same outcome measures (Morrison et al., 2020), in which dyadic partner ratings for first impression items, behavioral intentions, interaction quality, and closeness were all significantly related. However, Morrison et al. (2020) included autistic-autistic dyads and NA-NA dyads, in addition to the mixed dyads used in the current study. It may be the case that, consistent with a DEP framework (Milton et al., 2013), inter-partner agreement increases in interactions between people of a shared neurotype and declines within cross-diagnostic interactions.

Contrary to prediction, AAT largely did not affect trait evaluations made of and by autistic adults, nor did it affect participant assessments of interaction quality. While null findings should be interpreted with caution, several factors may account for the lack of training effects for some ratings. First, although a larger sample size may have revealed more effects, it is also likely that a brief, one-time presentation may be insufficient for eliciting the large-scale behavioral changes needed to improve interpersonal perceptions within these interactions. Indeed, previous work suggests that the effects of AAT modules, including the one used in this study, may affect explicit but not implicit biases (Bast et al., 2021; Jones et al., 2021). Implicit biases are automatic, unconscious forms of bias that can contribute to unfavorable judgments about groups of people, and as such, the persistence of these biases previously shown to be prevalent among NA participants toward autism (Dickter et al., 2020b), may have impacted behaviors and attitudes toward autistic interaction partners. Importantly, not all effects of training were beneficial. One unexpected result was that autistic and NA partners in the training condition rated one another as less trustworthy than in the control condition. Perhaps AAT influenced NA behavior in ways that were unappealing to autistic participants and/or raised suspicions among NA adults about their interaction partners. Alternatively, this could be a spurious finding related to using distinct participants in the two training conditions or to the lack of a pre-test/post-test design. The employed analyses did not implement a correction for family-wise error, so future work should examine whether this effect replicates.

While participants' diagnostic status was not disclosed, and only two autistic participants chose to disclose their diagnosis, it remains possible that social desirability biases in NA participants may have influenced the results of the study. Both groups were exposed to the possibility of an autistic conversation partner, but the autism-specific training video may have primed participants in this condition to expect an autistic partner, leading to more favorable ratings in this condition compared to a no-video control condition or a non-autism related control. If present, social desirability biases may represent a potential strength, as the methodology of this study maps onto how similar trainings may be administered in the real world, with participants aware that the training is designed to improve their interactions with autistic people. Regardless, training effects were not consistent across conditions, including a potentially negative finding of reduced trust in training condition participants, suggesting that results were not driven solely by social desirability biases. Additional research examining if and how demand characteristics influence training outcomes are encouraged.

Additionally, NA attitudes toward autism were not assessed prior to participation, so these may have differed between the two training groups, minimizing the potential benefits of AAT. The young adult NA sample included in the study also may have already been more familiar with, and accepting of, autistic differences than the general population (White et al., 2019). This is particularly relevant for the current NA sample given that it was drawn from a university with one of the largest number of autistic students in the United States (Hoffman, 2016). Further, the diagnostic status of participants was also not disclosed to conversation partners. While this lack of disclosure can be viewed as a methodological strength, producing fewer demand characteristics and more ecologically valid interactions, effects may have been larger if disclosure had occurred, as has been found in previous studies (Sasson and Morrison, 2019). Because the training was compared to a no-training condition, as opposed to an active control, it is difficult to ascertain whether any effects were specific to the training video used here, or rather an effect of training in general. Comparison to a more generic training, such as that used in our previous study (Jones et al., 2021), may illuminate the unique benefit of autism-specific training. Perhaps most importantly, the sample in this study consisted exclusively of White, self-identified males to control for confounding effects of cross-gender and cross-race interactions. Given the impact of gender (Milner et al., 2019; Lai and Szatmari, 2020) and racial biases (Giwa Onaiwu, 2020; Jones et al., 2020) on the experiences of autistic adults, the results of this study may have differed in important ways for a more diverse sample. Finally, participants were young adults with verbal IQs in the average range, so the impact of AAT may not generalize to the broader population of autistic people, including non-speaking people, those with an intellectual disability, or older adults. Therefore, while the current study offers proof of concept for analyzing the effects of NA training on improving interactions for autistic people, future work should consider how NA perceptions of, and behavior toward, autistic people intersect with other salient aspects of identity not examined here.

These limitations notwithstanding, this study provides further insight into the relational aspects that contribute to interaction difficulties between autistic and NA adults and offers some limited support for the benefits of AAT for NA adults. Autism acceptance training in this study was associated with a greater future interest in hanging out for both autistic and NA adults within dyads, but the benefits of training did not extend to other ratings, including evaluations of closeness and interaction quality. Findings also suggest that autistic and NA individuals may evaluate interactions differently, with NA individuals placing greater value on their partner's intelligence and social presentation. Therefore, while these findings offer some promise that the benefits of AAT may extend beyond the laboratory (Jones et al., 2021) into real-world settings and increase social interest between autistic and NA adults, more systematic changes are likely needed to bridge the communicative and interactive divide between autistic and NA adults.

## Data Availability Statement

The raw data supporting the conclusions of this article will be made available by the authors, without undue reservation.

## Ethics Statement

The studies involving human participants were reviewed and approved by The University of Texas at Dallas Institutional Review Board. The patients/participants provided their written informed consent to participate in this study.

## Author Contributions

KM, DJ, and NS led the design of the study, with contributions from AP and RA. DJ gathered and organized the data, with contributions from KM and KD. DJ conducted data analysis in collaboration with RA. DJ interpreted the data, with help from RA, AP, KD, and NS. DJ and NS wrote the manuscript, with contributions from AP, RA, and KD. All authors contributed to the article and approved the submitted version.

## Funding

This research was funded by the Texas Higher Education Coordinating Board's Autism Grant Program.

## Conflict of Interest

The authors declare that the research was conducted in the absence of any commercial or financial relationships that could be construed as a potential conflict of interest.

## Publisher's Note

All claims expressed in this article are solely those of the authors and do not necessarily represent those of their affiliated organizations, or those of the publisher, the editors and the reviewers. Any product that may be evaluated in this article, or claim that may be made by its manufacturer, is not guaranteed or endorsed by the publisher.
